# *Nfix* Expression Critically Modulates Early B Lymphopoiesis and Myelopoiesis

**DOI:** 10.1371/journal.pone.0120102

**Published:** 2015-03-17

**Authors:** Caitríona O’Connor, Joana Campos, Jason M. Osinski, Richard M. Gronostajski, Alison M. Michie, Karen Keeshan

**Affiliations:** 1 Paul O’Gorman Leukaemia Research Centre, Institute of Cancer Sciences, University of Glasgow, Glasgow, Scotland; 2 Genetics, Genomics and Bioinformatics Program and Department of Biochemistry, State University of New York at Buffalo, Buffalo, New York, United States of America; Emory University, UNITED STATES

## Abstract

The commitment of stem and progenitor cells toward specific hematopoietic lineages is tightly controlled by a number of transcription factors that regulate differentiation programs via the expression of lineage restricting genes. Nuclear factor one (NFI) transcription factors are important in regulating hematopoiesis and here we report an important physiological role of NFIX in B- and myeloid lineage commitment and differentiation. We demonstrate that NFIX acts as a regulator of lineage specification in the haematopoietic system and the expression of *Nfix* was transcriptionally downregulated as B cells commit and differentiate, whilst maintained in myeloid progenitor cells. Ectopic *Nfix* expression *in vivo* blocked early B cell development stage, coincident with the stage of its downregulation. Furthermore, loss of *Nfix* resulted in the perturbation of myeloid and lymphoid cell differentiation, and a skewing of gene expression involved in lineage fate determination. *Nfix* was able to promote myeloid differentiation of total bone marrow cells under B cell specific culture conditions but not when expressed in the hematopoietic stem cell (HSPC), consistent with its role in HSPC survival. The lineage choice determined by *Nfix* correlated with transcriptional changes in a number of genes, such as E2A, C/EBP, and Id genes. These data highlight a novel and critical role for NFIX transcription factor in hematopoiesis and in lineage specification.

## Introduction

Hematopoietic stem cells (HSCs) give rise to lineage restricted progenitor cells of the myeloid, lymphoid, and erythroid lineages through a series of commitment steps orchestrated by the expression of lineage restricting genes [1]. The nuclear factor one (NFI) protein family, also known as NF-I and CTF (CAAT box transcription factor), act as transcriptional activators and/or repressors of cellular and viral genes. In vertebrates, there are four closely related genes named NFIA, NFIB, NFIC, and NFIX [2]. They encode for proteins with a conserved N-terminal DNA-binding and dimerization domain and a C-terminal transactivation/repression domain, which exhibit a high variability due to extensive alternative splicing. NFI protein family members act as homo- and heterodimers and bind with high affinity to the palindromic consensus sequence 5′-PyTGGCA-N3-TGCCAPu-3′. NFI binding motifs were detected in promoters of genes expressed in different organs, including brain, lung, liver, intestine, muscle, connective tissue, skeletal elements and hematopoiesis. Thus, *NFI* genes have distinct functions depending on the cell type and target promoter context [2].

Recently *Nfia* was shown to regulate fate choice between erythrocytes and granulocytes in CD34^+^ human hematopoietic cell specification [3]. Its expression was abrogated to allow terminal granulocytic or monocytic differentiation via microRNA-223 and microRNA-424 respectively and C/EBPalpha and PU.1 interactions [4,5] indicating that *Nfia* silencing is a prerequisite for myelopoiesis. Furthermore, a transcriptome-wide approach revealed that *Nfia* induces an eythroid transcriptional program in both CD34^+^ and leukaemic K562 cells [6]. *Nfix* knockout mice die postnatally around p21 and have brain, intestine, and skeleton malformations [7,8] and *Nfix* deficient HSPCs fail to persist in long-term bone marrow engraftment upon transplantation [9]. Recently *Nfix* was shown to be one of 36 regulatory factors with relatively restricted expression in HSCs, that contributed towards converting a committed B cell to a myeloid cell [10]. These data indicate that *Nfi* proteins may act as putative drivers of lineage specification and perturbation.

In haematopoeitic cell maturation there are a number of transcription factors whose coordinated action are responsible for lineage specification and differentiation. For example, Pax5 can transcriptionally activate a B cell program while directly suppressing alternate lineage specific genes (e.g. myeloid-erythroid and T) [11]. Pax5^-/-^, E2A^-/-^, EBF and FOXO1 mice have arrested B cell development at the pro-B cell stage [12–15]. Indeed E2A^-/-^ mice have reduced HSCs with an increased proportion of cycling HSC and it was shown that E2A functions to promote the developmental progression of the entire spectrum of early hematopoietic progenitors [16,17]. Amongst other transcription factors known to play a role in myeloid and B lineage fate, PU.1 and C/EBPalpha are critical. High levels of PU.1 enforce myeloid development while low levels promote B cell differentiation [18]. In myeloid development, C/EBPalpha has a critical role in the commitment of mulitpotent progenitors into the myeloid lineage and knockout mice have a block in the transition from common myeloid progenitors (CMP) to granulocyte-macrophage progenitors (GMP) [19].

Here, we investigated the function of *Nfix* during hematopoietic differentiation and our data support the role of *Nfix* as a regulator of lineage fate in the hematopoietic system. *Nfix* modulated the differentiation of B cells and myeloid cells, effects that were seen in both overexpression and knockout systems. *Nfix* is transcriptionally downregulated as B cells differentiate whereas its expression is maintained in myeloid progenitor cells. Loss of *Nfix* expression perturbs myelopoiesis and enhances B lymphopoiesis. *Nfix*-mediated lineage determination was associated with transcriptional changes in a number of myeloid and lymphoid lineage specific genes. These data highlight a novel role for *Nfix* transcription factor in hematopoietic cell fate.

## Methods

### Animals

All experiments were approved and performed in accordance with local and home office regulations and the Experimental Animal Ethics Committees of University College Cork (B100/4097) and University of Glasgow (60/4512). *Nfix* ^-/-^ mice and litter matched WT controls were a gift from Richard Gronostajski.

### Bioinformatic Analyses

Expression profiles of the B-cell progenitors (GSE11110) [20] and for the human hematopoietic stem and progenitor cells, terminally differentiated cells, and intermediate state cells (GSE24759)[21] were downloaded from the Gene Expression Omnibus (http://www.ncbi.nlm.nih.gov/geo/). Using GenePattern (Version3.3.3) the Affymetrix CEL files for the B-cell progenitors were converted into a. gct file for analyses using the RMA method with quantile normalisation and background correct. Analyses of these datasets were carried out using GenePattern (Version3.3.3) and GraphPad Prism 5. GSEA was carried out using the GSEA program suite (version 2.07) available from the Broad Institute (http://www.broadinstitute.org/gsea/index.jsp) with gene sets obtained from the Molecular Signatures Database (MSigDB) (version 3.0.).

### Bone Marrow Transplant (BMT)

BMT experiments were performed as described previously. Briefly, 5FU (5mg) was administered intravenously to 6–8wk old C57Bl/6 wild type mice (WT). 4 days later whole BM was isolated, prestimulated overnight and retrovirally transduced *ex vivo* in the presence of IL-3 (10ng/ul), IL-6 (10ng/ul), and SCF (100ng/ul). Two 90 minute spinoculations were performed 24hours apart. 4 hours after the second spinoculation, 0.2–1x10^6^ unsorted cells were transplanted into lethally-irradiated C57Bl/6 recipients and maintained on antibiotics for 2 wks post-transplant. Engraftment was assessed by GFP expression in the peripheral blood 6 wks post-BMT.

### Cell Lines

32Dcl3 myeloid precursor cells and BA/F3 pro B cell lines [22] are both murine IL3-dependent cell lines maintained in RPMI, 10% FBS, 1% Penicillin/Streptomycin and 1% L-Glutamine supplemented with 10% WEHI conditioned media. OP9 stromal cells were kindly provided by J.C. Zuniga-Pflucker [23] were maintained in αMEM, 20% FBS, 1% Penicillin/Streptomycin, 1% L-Glutamine, 0.1% β-mercaptoethanol, 1% 100 mM sodium pyruvate and 1% HEPES (OP9 media).

### Constructs and retroviruses

A 1203 bp fragment encoding the entire murine *Nfix* cDNA was subcloned (+/- N or C terminus MYC tag) into pcDNA3.1/myc-HIS plasmid and MigR1 expressing GFP retrovirus.

### Flow Cytometry and Cell Sorting

Cell suspensions were stained on ice after blocking with rat/mouse IgG in PBS/2% FBS. Cells were sorted using a FACSAria-I (BDBiosciences) cell sorter. Analytical flow cytometry was performed on a LSRII or BDFACs Canto II (BDBiosciences) and analyzed using FlowJo software (Treestar). Cells were live gated using FSC/SSC, and single cells were analysed via FSC-H/FSC-A gating. Stem and progenitor populations are described in S1 Table. All antibodies used were obtained from eBioscience and are listed in S2 Table.

### Methylcellulose clonogenic assays

For total BM colony assays, BM cells were isolated from a C57Bl/6 mouse, and retrovirally-transduced with MigR1 and *Nfix ex vivo* in the presence of IL-3, IL-6, and SCF. Then, 20,000 GFP^+^ sorted cells were plated in triplicate in methylcellulose media (Methocult M3434; StemCell Technologies). For sorted populations total adult BM or FL cells from E14.5 mice were isolated and sorted for HSPCs, LMPPs, CMPs, GMPs and CLPs and transduced with MigR1 or *Nfix* then 24 hr later were plated at 5000 (HSPC), 25,000 (LMPP), 40,000 (CMP), 60,000 (GMP) or 12,000 (CLP) in methylcellulose media. Colonies with >50 cells were scored and assessed at 12–14 d.

### OP9 cultures

FL was isolated from E14.5 C57Bl/6 embryos and a cell suspension was prepared. Cells were incubated overnight in OP9 media supplemented with 20 ng/ml mSCF, 10 ng/ml IL6 and 10 ng/ml IL3. Cells were then retrovirally-transduced with MigR1 or *Nfix* retrovirus. 24 hr post-transduction these cells were either sorted for HSPCs and pro-B cells or plated without sorting onto OP9 cells in complete MEM media supplemented with 5 ng/ml Flt3 and 1 ng/ml IL7. Media was refreshed every 2 d and cells were replated to fresh OP9 cells every 4 d for 12–16 d, at which point the cells were removed and stained in PBS with PerCPCy5.5-anti-B220, APC-anti-CD19, AlexaFluor700-anti-CD11b, eFluor450-anti-Gr-1, biotinylated anti-CD45 (to exclude OP9 cells) with streptavidin-PeCy7 secondary antibody. Cells were acquired on a LSRII or BDFACS CantoII and analysed using FlowJo V7.6.5.

### Quantitative RT-PCR

RNA was isolated from 32D and BA/F3 cells (transduced with control MigR1 or *Nfix* retrovirus and sorted for GFP), and BM cells, using the RNEasy kit (Qiagen), then digested with DNaseI and used for reverse transcription according to the manufacturer’s instructions (Superscript II kit, Invitrogen). RT-PCR was performed on the ABI Prism 7900 sequence detection system (Applied Biosystems). The ∆∆CT method was used to calculate the relative level of gene expression in each sample. High-throughput qPCR was performed on the 48.48 Dynamic Array IFC system (Fluidigm). Primer sequences are listed in S3 Table. Specific target pre-amplification was carried out. All reactions were carried out in triplicate. Expression levels of the target genes were normalized relative to the mean of the reference genes ABL, GUSB, B2M and either TBP and HPRT1, or ENOX2 and RNF20. Relative mRNA levels were calculated using the 2 -∆∆CT method.

## Results

### Altered hematopoiesis in *Nfix* chimeric mice

**Fig 1:**
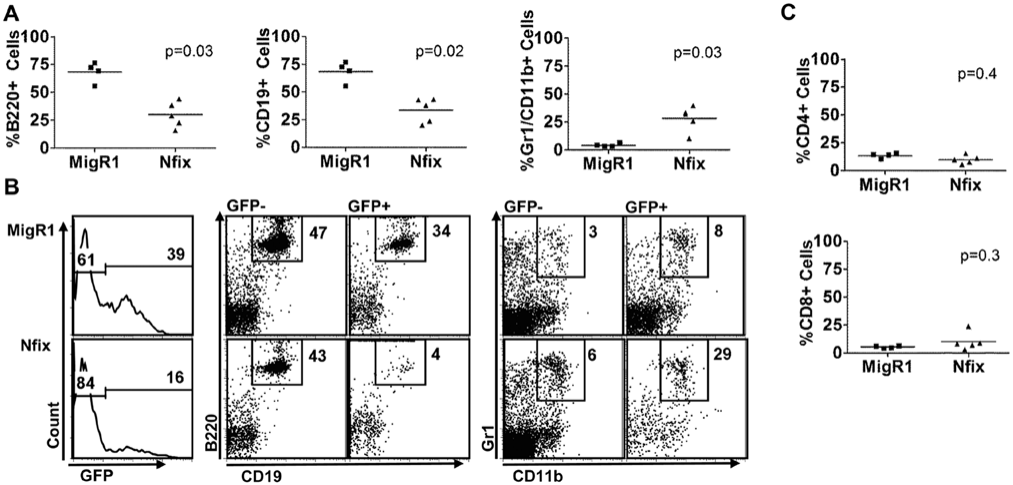
Decreased B cells and increased myeloid cells in *Nfix* expressing cells in the periphery. C57Bl/6 mice were reconstituted with BM cells transduced with control MigR1 or *Nfix* vectors. **(A)** Graph of percentage number of GFP^+^ B cells (B220^+^, CD19^+^), myeloid cells (Gr-1^+^CD11b^+^), and **(C)** T cells (CD4^+^, CD8^+^) in the PB of MigR1 and *Nfix* chimeric animals 6 weeks post-transplant. **(B)** Representative flow cytometric analysis of PB cells in MigR1 and *Nfix* chimeric animals, 10 wk post-transplant, showing engraftment of GFP (left panel), B220^+^CD19^+^ B cells in the GFP^-^ and GFP^+^ fractions (middle panel, percentages given) and the Gr-1^+^CD11b^+^ myeloid cells in the GFP^-^ and GFP^+^ fractions (right panels, percentages given). Results are representative of 2 independent BMT experiments.

**Fig 2:**
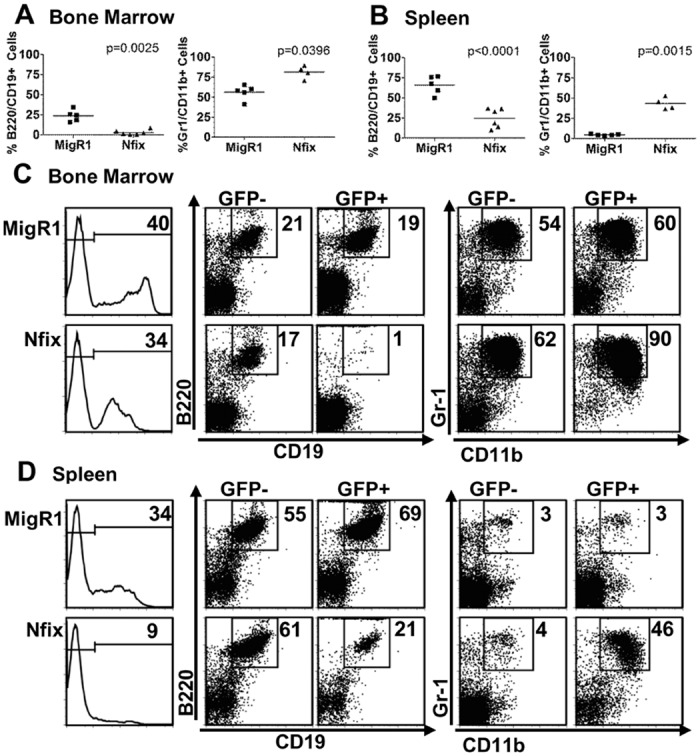
Decreased B cells and increased myeloid cells in *Nfix* expressing cells in the bone marrow and spleen. C57Bl/6 mice were reconstituted with BM cells transduced with control MigR1 or *Nfix* vectors. Graph of percentage of B cells (left, B220^+^CD19^+^) and myeloid cells (right, Gr-1^+^CD11b^+^) in the **(A)** BM and **(B)** Spleen of MigR1 and *Nfix* chimeric animals (GFP^+^) 10 wks post-transplant. Representative flow cytometric analysis in MigR1 and NFIX chimeric animals, 10 wk post transplant, showing GFP engraftment (left panel), B220^+^CD19^+^ B cells (middle panel, percentages given) and Gr-1^+^CD11b^+^ myeloid cells (right panel) in the GFP^-^ and GFP^+^ fractions of the bone marrow (C) and spleen (D). Results are representative of 2 independent BMT experiments.

To investigate a role for *Nfix* in haematopoietic cells, lethally-irradiated C57BL/6 mice were reconstituted with retroviral control (MigR1) or *Nfix* expressing (MigR1-*Nfix*) vector transduced hematopoietic progenitors. At 6–10 wk post bone marrow transplant (BMT), reconstitution within the myeloid, B and T cell compartments was assessed in peripheral blood (PB). The percentage of both B220^+^ and CD19^+^ B cells was significantly reduced in the GFP^+^ population of *Nfix* mice compared to MigR1 (Fig. 1A and B) whereas there was no change in the PB CD4^+^ and CD8^+^ T cell compartment (Fig. 1C). The reduction noted in the percentage of the B cell compartment was complemented by a significant increase in the percentage of GFP^+^Gr-1^+^CD11b^+^ myeloid cells in the periphery of *Nfix* chimeric mice (Fig. 1A and B). These data suggest that enforced expression of *Nfix* results in the perturbation of peripheral myeloid and B cell populations. A more pronounced effect was evident in the BM and spleen of *Nfix* chimeras (Fig. 2). The percentage of GFP^+^B220^+^CD19^+^ B cells was dramatically decreased in the BM and spleen, whereas the percentage of GFP^+^Gr-1^+^CD11b^+^ myeloid cells was significantly increased in the BM and spleen (Fig. 2A-C). Analysis of the GFP percentage in B cell and myeloid populations in the periphery, BM and spleen revealed that the block in B cell and increase in myeloid cells was likely due to the autonomous effect of *Nfix* expression in the cell and that the defective B lymphopoiesis was not due to the myeloid hyperplasia (data not shown).

### *Nfix* expression decreases during B cell development

**Fig 3:**
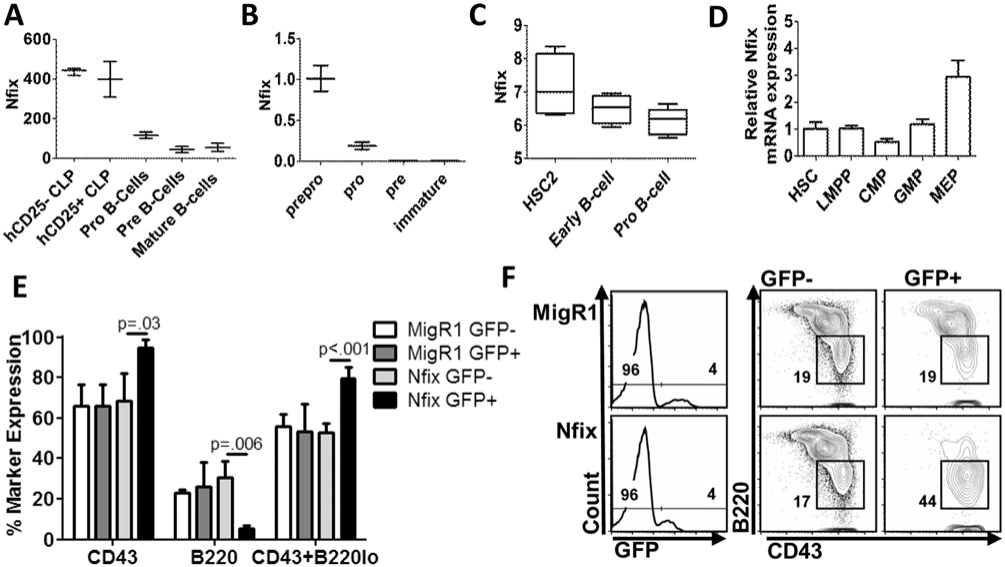
Nfix expression in stem and progenitor populations. **(A)** Analysis of *Nfix* expression during murine B cell differentiation using the B-cell lineage microarray dataset (GSE11110)[41]. Error bars represent max and min *Nfix* expression values. **(B)** Quantitative RT-PCR analysis of *Nfix* expression in murine B cell populations fractionated by flow cytometry. Data are representative of three independent experiments, performed in duplicate. Error bars denote ±SD. **(C)** Analysis of *NFIX* expression in human B cell development from the human hematopoiesis microarray dataset (GSE24759) (21). Plots represent raw values, error bars represent max and min values. **(D)** Analysis of *Nfix* expression by qRT-PCR in murine stem and progenitor cells isolated from bone marrow. **(E)** Graph of percentage expression of CD43^+^, B220^+^, and B220^lo^CD43^+^ populations in BM cells from chimeras established with MigR1 or *Nfix* progenitors 8–10 wks previously. Error bars denote +/- SD of 2 independent experiments (n = 3). **(F)** Representative flow cytometric analysis, with gates and percentages showing B220^lo^CD43^+^ populations (early B cells) in GFP^-^ and GFP^+^ populations.

As we saw a block in the development of B220^+^CD19^+^ B cells in the BM, spleen and PB, we sought to determine the endogenous expression of *Nfix* during B lymphopoiesis. We analysed the publically available microarray dataset of mouse B cell lineage populations accessed through the GEO database [20]. This dataset included hCD25^-^ and hCD25^+^ CLP (Lin^-^B220^-^CD19^-^CD127^+^Flt3^+^Sca1^low^Kit^low^)(hCD25 under the control of the B-lineage restricted Igll1 promoter), pro-B (CD19^+^AA4.1^+^CD43^low^), pre-B (CD19^+^B220^+^CD43^-^IgM^-^) and mature spleen (IgM^+^CD19^+^) B cells. Endogenous *Nfix* expression was dramatically reduced at the pro-B cell stage of B lymphopoiseis compared to the CLP populations, and further decreased as the B cells differentiate to pre-B and mature spleen B cells (Fig. 3A). We next performed qRT-PCR on B cell progenitor fractions sorted from WT C57Bl/6 mice. *Nfix* mRNA expression was assessed in pre-pro B (B220^+^CD43^+^CD19^-^), pro-B (B220^+^CD43^+^CD19^+^), pre-B (B220^+^CD43^-^CD19^+^IgM^-^), and immature B (B220^+^CD43^-^CD19^+^IgM^+^). Consistent with the microarray analysis, *Nfix* expression decreased after the pre-pro-B cell stage and continued to decrease to the immature B cell stage (Fig. 3B). As a control, gene expression analysis of lineage specific transcription factors in the microarray dataset and in mRNA from sorted murine populations revealed consistent results (S1 Fig.). We next assessed whether this pattern of *NFIX* expression was evident in human B cell subsets. Using the microarray dataset from human cell compartments accessed through the GEO database [21] we assessed *NFIX* expression in the HSC2 (Lin^-^CD38^-^CD34^+^), early B-cell (CD34^+^CD10^+^CD19^+^) and pro-B cell (CD34^-^CD10^+^CD19^+^) compartments. There was a decrease in *NFIX* expression from the HSC2 compartment of human cells to the pro-B cell compartment (Fig. 3C). *Nfix* mRNA expression was further assessed in HSPC through to myeloid progenitor populations (HSPC (Lin^-^cKit^+^Scat1^+^Flt3^-^), LMPP (Lin^-^cKit^+^Scat1^+^Flt3^+^), CMP (Lin^-^cKit^-^CD34^int^CD16/32^int^), GMP (Lin^-^cKit^-^CD34^+^CD16/32^+^) and MEP (Lin^-^cKit^-^CD34^lo^CD16/32^lo^)). *Nfix* expression was not downregulated in myeloid restricted progenitor populations (Fig. 3D). We observed similarities in *NFIX* expression using HemaExplorer platform (data not shown) [24]. These data show that *NFIX* expression decreased during both murine and human B cell lineage differentiation. To further support this, a detailed B cell development flow cytometric analyses was performed in *Nfix* chimeras *in vivo*. Consistent with our bioinformatics analyses showing a decrease in *Nfix* expression at the early stages of B cell stage of development, we saw an accumulation of the early B cell marker CD43 (which is normally turned off at the pre-B cell stage) and B220^lo^CD43^+^ cells, and a concomitant decrease in the percentage of B220^hi^ and CD43^-^ B cells in *Nfix* chimeric animals as compared to MigR1 controls (Fig. 3E and F). These data demonstrate that ectopic expression of *Nfix* resulted in a block in early B-cell development *in vivo*, consistent with the stage when endogenous *Nfix* is downregulated.

### *Nfix* expression favors myelopoiesis over B cell lymphopoiesis

**Fig 4:**
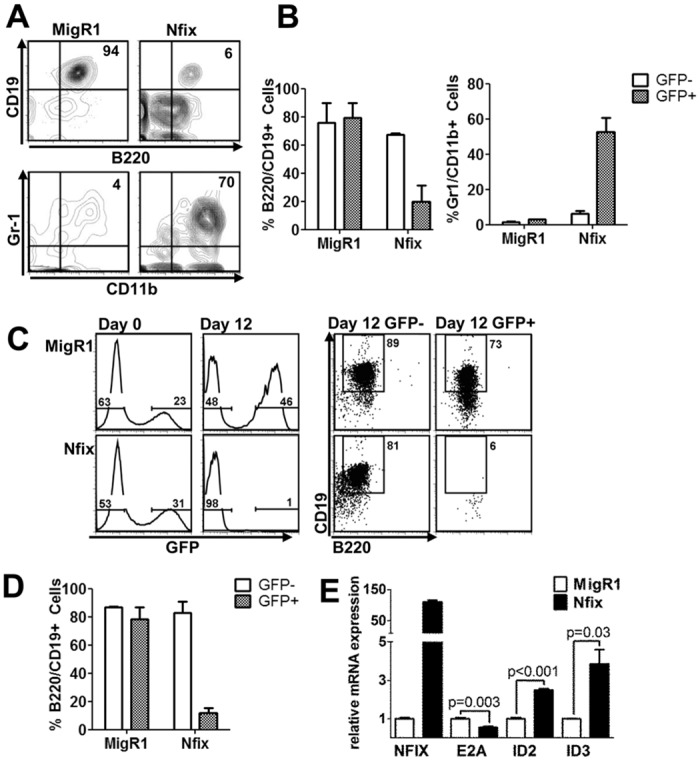
*Nfix* blocks B cell differentiation in vitro in favour of myelopoiesis. **(A)** Total FL was transduced with control MigR1 or *Nfix*, plated on OP9 cells and analyzed by flow cytometry on day 16. FACS plots of GFP^+^ B cells (B220^+^CD19^+^, top panels) and GFP^+^ myeloid cells (CD11b^+^Gr-1^+^, lower panels) representative of 3 replicates from 2 independent experiments. **(B)** Graph of average percentage of B220^+^CD19^+^ (left) and CD11b^+^Gr-1^+^ (right) from (A). Error bars denote +/- SD of 2 independent experiments (n = 3). Graph shows a statistically significant decrease in *Nfix* derived B220^+^CD19^+^ cells (p = 0.02) and a significant increase in *Nfix* derived CD11b^+^Gr-1^+^ cells (p = 0.01) when compared with MigR1 controls. **(C)** E14.5 FL HSPCs (Lin^-^Sca1^+^cKit^+^Flt3^-^) transduced with control MigR1 or *Nfix* retrovirus were plated on OP9 cells (Day 0) and analysed by flow cytometry on day 12 with FACs plots of GFP expression (left histograms) and GFP^-^ and GFP^+^ B220^+^CD19^+^ cells (right dot plots) representative of 2 independent experiments. **(D)** Graph of average percentage of B220^+^CD19^+^ cells from (C), error bars denote +/- SD of 2 independent experiments. Experiment shows significant decrease in *Nfix* expressing B cells compared with control MigR1 (p = 0.01) **(E)** Total BM cells were sorted for MigR1 and *Nfix* expression 24 hr post transduction and qRT-PCR was performed. Graphical presentation of relative mRNA expression of *Nfix*, E2A, ID2 and ID3 genes and presented relative to control MigR1 cells. Error bars denote +/- SD of 3 technical replicates. Data representative of 2 biological replicates.

To further investigate the block in B cell and increase in myeloid cell development we observed *in vivo*, we carried out *in vitro* differentiation assays using the BM stromal OP9 cell line that supports B and myeloid cell development depending on the cytokines used. Unsorted E14.5 fetal liver (FL) cells were retrovirally transduced with MigR1 or MigR1-*Nfix* and cultured on OP9 cells supplemented with 5 ng/ml FLT3 and 1 ng/ml IL7 to support B cell development. MigR1 expressing control FL cells robustly developed B cells after 16 days co-culture whereas in contrast, *Nfix* expressing FL cells were unable to develop B220^+^CD19^+^ B cells. Furthermore, *Nfix* expressing FL cells efficiently differentiated into CD11b^+^Gr-1^+^ myeloid cells in this system, despite being in the presence of B cell permissive cytokines (Fig. 4A and B). GFP percentages throughout the culture revealed that as B cells differentiate in this culture, the GFP percentage declines (S2A Fig.). To determine whether *Nfix* expression could drive myelopoiesis and block B lymphopoiesis from HSPCs (Lin^-^Sca1^+^cKit^+^Flt3^-^), we retrovirally transduced purified FL-HSPC with MigR1 and *Nfix* and cultured on OP9 cells supplemented with 5 ng/ml FLT3 and 1 ng/ml IL7. *Nfix* overexpression in purified HSPCs abrogates B cell development in this OP9 culture system (Fig. 4C and D, S2B Fig.). *Nfix* overexpression is however not toxic to committed pro B cells, as BA/F3 cells overexpressing *Nfix* do not have any evidence of altered cell growth, proliferation or apoptosis (S2C–G Fig.). To assess the molecular changes involved in the perturbation of lineage specificity in *Nfix* expressing cells, we carried out gene expression analysis on a number of lineage specific targets (Fig. 4E and S3 Table). It has been proposed that upregulation of ID genes and subsequent E-protein inhibition are involved in myeloid lineage commitment while shutting off the lymphoid fate as cells transit from the LMPP compartment [25]. Our analysis showed that ectopic *Nfix* expression in BM cells led to significant decrease in E2A, and concomitant increase in ID2 and ID3 (Fig. 4C). These gene expression changes correlate with the *Nfix*-mediated lineage perturbations *in vitro* and *in vivo*.

**Fig 5:**
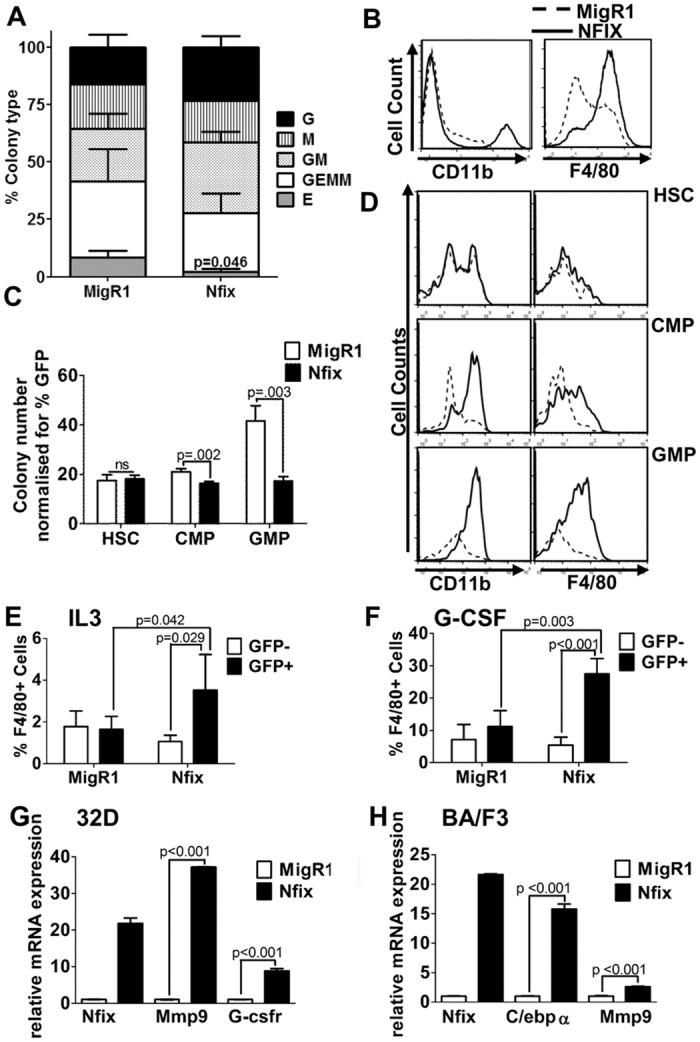
Efficient differentiation of myeloid cells in *Nfix* expressing cells. **(A)** MigR1 and *Nfix* transduced total BM cells were plated in methylcellulose media (M3434) and colonies scored after 9–11 days according to morphological criteria. The mean percentage of erythroid (E), granulocyte/erythrocyte/monocyte/megakaryocyte (GEMM), granulocyte/monocyte (GM) macrophage (M) and granulocyte (G) colonies are shown from 3 independent experiments +/- SEM. A statistically significant decrease in CFU-EE colonies indicated. **(B)** Representative flow cytometric analysis of total cells from colony assay in (A). **(C)** Graph of methylcellulose colony numbers from HSPCs, CMPs and GMPs sorted and transduced with control MigR1 or *Nfix*. Colonies were counted on day 12 and graph represents mean of triplicate plates normalized to GFP expression +/- SD. **(D)** Representative flow cytometric analysis of cells from colony assay in (C). 32D cells transduced with MigR1 or *Nfix* maintained in either IL3 **(E)** or G-CSF **(F)** for 5 days, and analyzed for F4/80 expression by flow cytometry. The mean percentages of 3 independent experiments are shown +/- SD. **(G)** 32D cells and **(H)** Ba/F3 cells transduced with MigR1 and *Nfix* and qRT-PCR performed. Graphical presentation of relative mRNA expression of *Nfix*, MMP9, G-CSFr and C/EBPalpha genes and presented relative to control MigR1 cells. Error bars denote +/- SD of 3 technical replicates. Data representative of 2 biological replicates.

Myeloid differentiation was further investigated by plating MigR1 and *Nfix* expressing total BM cells, and purified HSPCs, CMP and GMP populations (immunophenotype markers in supplementary methods) in methylcellulose media that supports optimal growth of erythroid progenitors (BFU-E), granulocyte-macrophage progenitors (CFU-GM, CFU-M, CFU-G) and multi-potential granulocyte, erythroid, macrophage, megakaryocyte progenitors (CFU-GEMM) cells (M3434 methocult). *Nfix* overexpression in bulk BM cells did not change total colony number or the number of cells per colony type. However there is a clear trend towards lower numbers of immature NFIX derived colonies compared with MigR1 (CFU-E and CFU-GEMM) with a concomitant increase in more mature myeloid colonies (CFU-GM and CFU-G) (Fig. 5A). This was evident by an increase in CD11b and F4/80 marker expression detected by flow cytometry (Fig. 5B). In HSPCs, there was no significant difference in the number of colonies derived from *Nfix* expressing HSPCs when compared with control, however, there was a significant decrease in the number of colonies formed from CMP and GMP cells expressing *Nfix* (Fig. 5C). Flow cytometric analysis of the colonies following 12–14 days in culture revealed a pronounced increase in the number of CD11b^+^ and F4/80^+^ cells (markers of mature myeloid cells) in CMP and GMP colonies when compared with control (Fig. 5D). The decrease in colony number and in increase CD11b^+^ and F4/80^+^ indicate that *Nfix* drives mature myeloid differentiation and these effects are pronounced at the CMP stage of development. To further support the role of *Nfix* in myeloid lineage differentiation, we expressed *Nfix* in the IL-3 dependent 32D myeloid cell line and assessed the effect on myeloid cell surface differentiation markers. There was a statistically significant increase in myeloid differentiation as assessed by the expression of F4/80 in the presence of IL3 alone upon *Nfix* expression, which was further enhanced in the presence of the myeloid-permissive cytokine G-CSF (Fig. 5E and F). Gene expression analysis of 32D and BA/F3 sorted for *Nfix* expression revealed that *Nfix* significantly increased the expression of the myeloid specific genes C/EBPalpha and target gene GCSF-R and MMP9 (Fig. 5G and H). These data strongly suggest that ectopic expression of *Nfix in vitro* and *in vivo* biases hematopoietic progenitors to the myeloid lineage and suppresses lymphoid development.

### Loss of *Nfix* expression promotes B lymphopoiesis while impairing myelopoiesis

**Fig 6:**
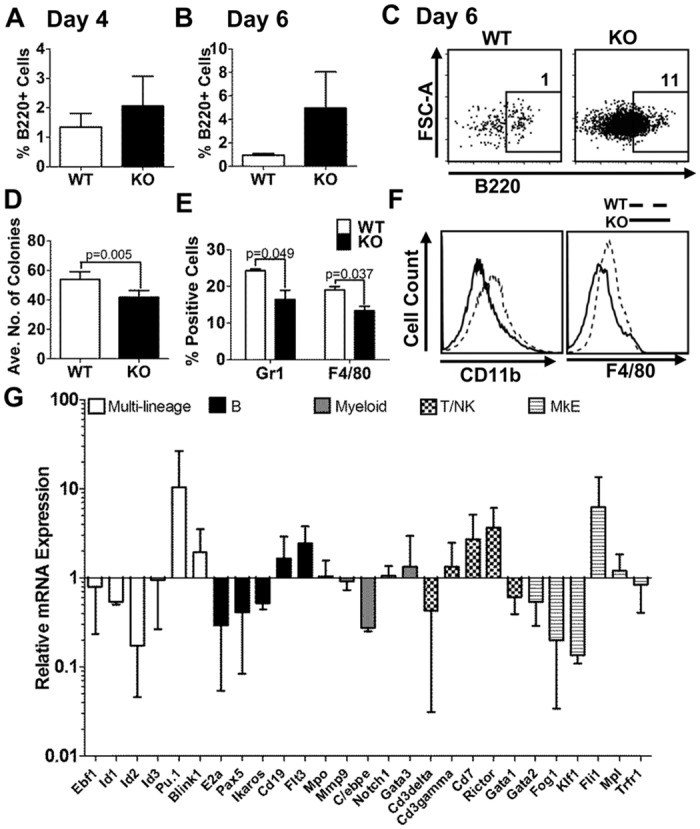
Loss of *Nfix* perturbs myelopoiesis while driving B cell differentiation. Total BM from WT or *Nfix* deficient mice was plated onto OP9 cells and analysed by flow cytometry on day 4 **(A)** and day 6 **(B)**. Bar chart shows the mean percentages of B220^+^ cells from 3 independent experiments +/- SD. **(C)** Representative flow cytometric analysis of cells expressing B220 at day 6. **(D)** Total BM from WT or *Nfix* deficient mice was plated in methylcellulose (M3434). Colonies were counted after 11 days. Bar chart represents the mean number of colonies from 2 independent experiments +/- SD. **(E)** Bar chart shows the mean percentages of Gr1 ^+^ and F4/80^+^ cells from 2 independent experiments +/- SD. **(F)** Representative flow cytometric analysis of cells from colony assay which were stained with Gr-1 and F4/80. **(G)** Graphical presentation of relative mRNA expression of indicated genes assessed using high-throughput qPCR on the 48.48 Dynamic Array IFC system (Fluidigm). Bars represent the average of 3 biological *Nfix^-/-^* replicates normalized to WT control, and error bars denote +/- SD.

To investigate the physiological relevance of *Nfix* in myeloid and lymphoid lineage specification, we isolated BM from *Nfix* ^-/-^ neonate mice and cultured it on OP9 cells. There was a marked increase in cells expressing B220 after 4 and 6 days in culture compared with WT littermate control cells (Fig. 6A-C). Myeloid differentiation of *Nfix* deficient cells was assessed in methylcellulose colony assays. *Nfix* deficient BM cells formed significantly fewer colonies than their WT counterparts (Fig. 6D), and expressed significantly lower percentages of mature myeloid markers CD11b, Gr1 and F4/80 (Fig. 6D-F). These data indicate that loss of *Nfix* perturbs normal myeloid differentiation. Gene expression analysis by qRT-PCR reveals that *Nfix* deficient BM cells have a marked disruption in the expression of key transcription factors involved in lineage fate determination (Fig. 6G). Of particular interest is the downregulation of Id genes and MMP9 (the inverse of our overexpression data), and an upregulation of CD19 and Pu.1 in *Nfix* deficient cells, supporting a role of *Nfix* in cell lineage fate. Taken together, these loss of function data show that loss of *Nfix* in the BM leads to altered hematopoiesis by enhancing B cell development while disrupting myelopoiesis, and that this effect is mediated by changes in key transcription factors associated with lineage fate determination.

## Discussion

We report an important role of the transcription factor *Nfix* in hematopoietic cell fate, specifically in B- and myeloid lineage differentiation. Our studies indicate that *Nfix* expression is downregulated as the cells become committed to the B cell lineage, and loss of *Nfix* expression enhances B cell lineage fate, concomitant with skewed myelopoiesis and altered expression of lineage fate genes. Enforced *Nfix* expression prevents early B cell development and favors myeloid differentiation *in vitro* and *in vivo* which correlates with an alteration in lineage-specific commitment genes of the B and myeloid lineages.

We noted higher *Nfix* mRNA expression in the early B cell progenitor populations in both mouse and human hematopoietic cells and downregulation of its expression as cells commit and mature to the B cell lineage. Our data shows that upon ectopic expression of *Nfix in vivo*, B cell development is severely impaired in the BM and spleen of chimeric animals. Although some CD19^+^B220^+^ B cells were still present in the spleen of *Nfix* chimeric animals, there was almost complete block in CD19^+^B220^+^ B cell production in the BM. These data suggest that upon continued expression of *Nfix*, B cell development in the BM is impaired. In accordance with the downregulation in *Nfix* mRNA expression to pro-B cell stage of differentiation, accumulation of B220^lo^CD43^+^ early B cells in the BM of *Nfix* chimeric animals was observed. Additionally, using the *in vitro* OP9 co-culture system, enforced *Nfix* expression in the HSPC compartment led to an almost complete absence of *Nfix* expressing cells at the end of the culture period. The very small percentage of surviving *Nfix* expressing cells derived from HSPCs do not express CD19 yet do express B220^lo^ consistent with the block at an early stage of development seen *in vivo*. *Nfix* surviving cells were observed when the transduced population included more myeloid committed progenitors even when cultured on OP9 cells in B cell cytokine conditions. In this case, *Nfix* expressing cells were of the myeloid lineage. The fact that this culture system contained no myeloid specific cytokines and generates an environment that efficiently produces B cells as seen by control cultures, provides strong evidence that *Nfix* has a functional role in lineage fate.

Transcriptional lineage priming of hematopoietic stem and progenitor cells has been shown to define their lineage potential [26]. The commitment of hematopoietic progenitors to the B cell lineage and their development to mature B cells depends on the combined activities of the transcription factors E2A, EBF, and Pax5 [27]. The earliest B cell progenitors express a lineage restricted marker B220, together with CD43 [28]. The progenitors are mostly B lineage committed, even in the absence of detectable CD19 surface expression (27) and PAX5 [29]. E proteins are well known to be crucial for lymphopoiesis [14] however recently it was shown that HSCs and LMPPs from E2A^-/-^ mice show an increase in proliferation accompanied by efficient myeloid- but not lymphoid cell differentiation [16]. Indeed, E2A-deficient HSCs fail to maintain the HSC pool and the entire spectrum of early hematopoietic progenitors [17]. E-proteins are antagonized by the Id proteins, which contain an HLH motif but lack a DNA binding region [30]. Furthermore, it has been documented that the downregulation of both Id2 and Id3 is an essential event in B-lineage specification although the loss of either factor was not sufficient to promote B cell development [31]. We observed significant changes in E2A, Id2 and Id3 in *Nfix*-deficient cells and upon ectopic *Nfix* expression. This perturbation in the levels of E2A and Id gene expression are consistent with a block in B cell lineage commitment and differentiation and the efficient myeloid cell production observed. The modulation of Id expression can induce a reversal of B lineage commitment [32] and Id-mediated inhibition of E proteins was shown to be involved in myeloid lineage commitment [25] suggesting that the balance between Id and E proteins regulates myeloid-versus-lymphoid lineage decisions.

Cell fate decisions during hematopoiesis are governed by lineage-specific transcription factors e.g. PU.1 and C/EBP family members. PU.1 transcription factor has a well-documented central role controlling myeloid and early B and T-cell development and PU.1 expression levels are important in the determination of lineage fate [33,34]. Recently it was shown that PU.1 expression is regulated differentially in B cells and myeloid cells through differential association with cell-type specific transcription factors and a balance of sense and antisense RNAs at one of its upstream regulatory elements [35,36] and through cell cycle duration effects [37]. The modulation of PU.1 seen in *Nfix*-deficient cells is consistent with the role of PU.1 in myeloid versus B lineage cell fate. It has previously been shown that CEBPA loss-of-function mutations decrease myeloid priming of HSCs while simultaneously impairing myeloid-lineage commitment [38]. In addition, C/EBPs are known to be regulated by Runx1 [39] and to positively regulate PU.1 expression [40] in myeloid lineage fate choices. A recent study identified 36 factors that could reprogram a committed B cell into a myeloid cell and *Nfix* was one of these factors [10]. Our data show that alone, *Nfix* can modulate the fate of a stem and progenitor cell toward the B and myeloid lineage. *Nfix* can thus be hypothesized to act as a molecular switch involved in lineage instruction and commitment. Whether this occurs due to lineage priming in the stem cell compartment or due to antagonistic or synergistic activity in a committed progenitor cell is not yet known.

The data presented here indicate that the failure to downregulate *Nfix* would be detrimental to B cell production and favor myelopoiesis. Collectively, these data suggest that *Nfix* expression at early stages of development (HSPCs) can modulate the myeloid-versus-lymphoid divergent cell fate, potentially via the direct modulation of downstream transcriptional targets.

## Supporting Information

S1 Fig Gene expression in human and murine B cell progenitor populations. (A) B-cell and myeloid cell gene expression in the B-cell progenitor microarray data. Max gene expression data was calculated for each B-cell progenitor population from the B-cell progenitor microarray dataset (GSE11110). Microarray data was collapsed to max probe expression for each gene using the Collapse Dataset suite in GenePattern. Values for each progenitor cell type were then plotted using GraphPad. Error bars represent max and min values for each cell type. (B) Quantitative RT-PCR analysis of B cell and myeloid cell specific gene expression in sorted murine B cell populations fractionated by flow cytometry. Relative levels of CEBPA, Il7R, NOTCH1, PAX5, SFPI1, EBF1, CD19 and BLNK expression in pre-pro, pro, pre and immature B cell populations. 18S was used as an endogenous control. Each gene/18s ratio in pre-pro B cell progenitors was normalized to 1.
(TIF)

S2 Fig Ectopic *Nfix* expression does not effect cell growth or viability but in B cell growth conditions BM cells expressing *Nfix* are lost over time. (A) Tracking GFP expression in MigR1 and *Nfix* expressing total FLCs over 16 days in culture. Graph represents the average +/- SD of 2 independent experiments (3 technical replicates). (B) Graph of the average GFP expression in MigR1 and *Nfix* expressing FLC HSPCs after 0 and 12 days in OP9 co-culture. Error bars denote +/-SD of 2 biological replicates. (C) Tracking GFP expression in MigR1 and *Nfix* expressing BaF/3 cells over 5 days in culture, showing mean of 3 technical replicates +/- SD. Graph is representative of 2 independent experiments. (D) Graph of 5 days of cell growth of GFP sorted BaF/3 cells transduced with either MigR1 or *Nfix*. Each point represents the average number of cells from 2 independent experiments. Error bars denote +/- SD. (E) Tracking Baf/3 cell division after 5 days in culture as shown by dilution of Cell Trace Violet in GFP expressing cells. MigR1 and *Nfix* overexpressing cells are compared to unstained control and Day 0 cells as positive and negative controls respectively. Graph is representative of 2 independent experiments. (F) Representative FACs plot of the expression of apoptotic markers AnnexinV and DAPI in MigR1 or *Nfix* transduced, GFP sorted BaF/3 cells, 4 days after transduction. (G) Graph of the average percentage of GFP sorted BaF/3 cells expressing DAPI, AnnexinV and live cells (double negative for DAPI and AnnexinV), from 2 independent experiments. Error bars denote +/- SD.
(TIF)

S1 Table Sorting stem and progenitor populations. (PDF)

S2 Table FACs Antibodies (eBioScience). (PDF)

S3 Table Primer Sequences (rtPCR & Fluidigm). (PDF)
