# Supplementary figures and images for: Nfix Expression Critically Modulates Early B Lymphopoiesis and Myelopoiesis
Source: PLoS One. 2015 Mar 17;10(3):e0120102. doi: 10.1371/journal.pone.0120102 (PMC4363787; doi:10.1371/journal.pone.0120102)

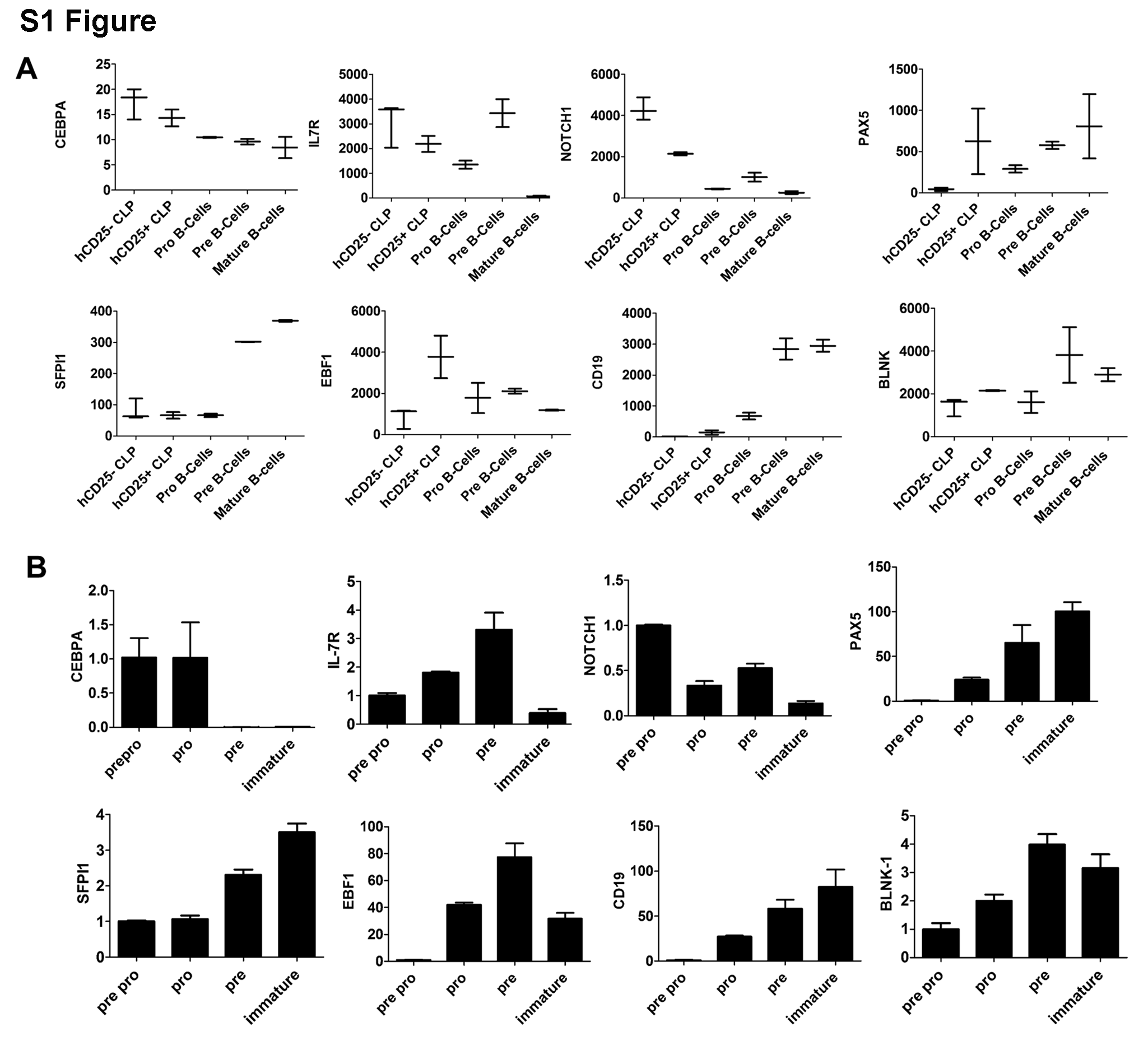

Supplement: S1 Fig — (A) B-cell and myeloid cell gene expression in the B-cell progenitor microarray data. Max gene expression data was calculated for each B-cell progenitor population from the B-cell progenitor microarray dataset (GSE11110). Microarray data was collapsed to max probe expression for each gene using the Collapse Dataset suite in GenePattern. Values for each progenitor cell type were then plotted using GraphPad. Error bars represent max and min values for each cell type. (B) Quantitative RT-PCR analysis of B cell and myeloid cell specific gene expression in sorted murine B cell populations fractionated by flow cytometry. Relative levels of CEBPA, Il7R, NOTCH1, PAX5, SFPI1, EBF1, CD19 and BLNK expression in pre-pro, pro, pre and immature B cell populations. 18S was used as an endogenous control. Each gene/18s ratio in pre-pro B cell progenitors was normalized to 1. (TIF) [file pone.0120102.s001.tif]

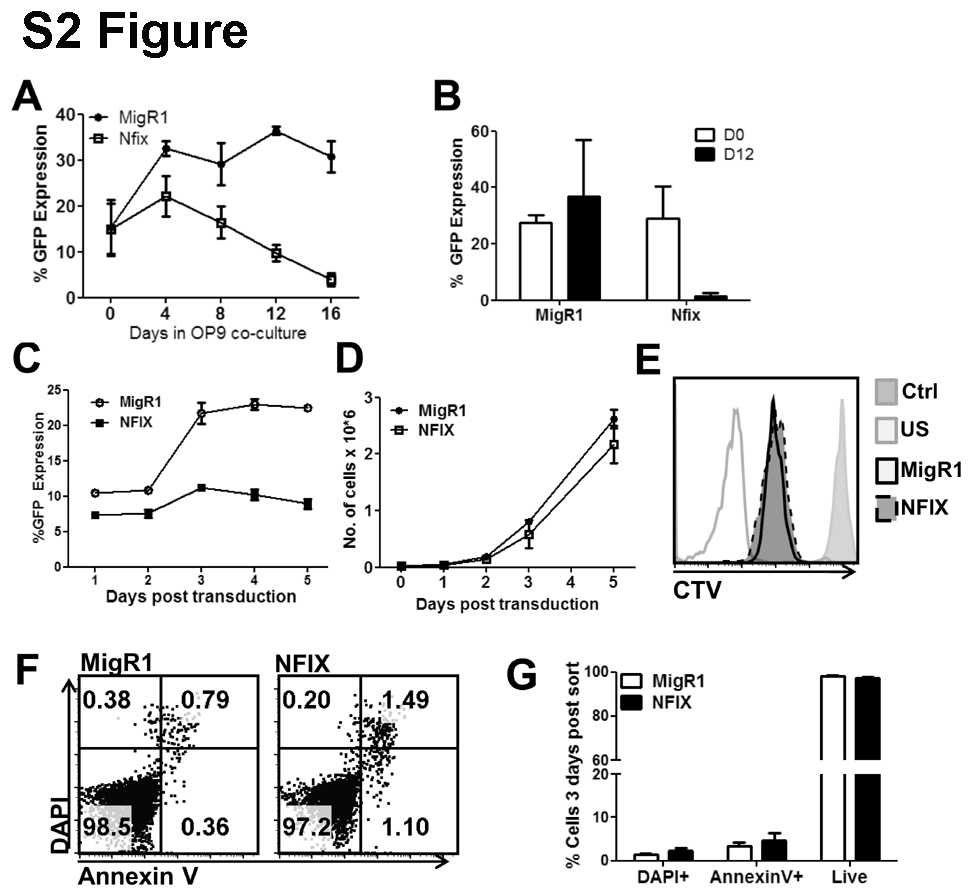

Supplement: S2 Fig — (A) Tracking GFP expression in MigR1 and Nfix expressing total FLCs over 16 days in culture. Graph represents the average +/- SD of 2 independent experiments (3 technical replicates). (B) Graph of the average GFP expression in MigR1 and Nfix expressing FLC HSPCs after 0 and 12 days in OP9 co-culture. Error bars denote +/-SD of 2 biological replicates. (C) Tracking GFP expression in MigR1 and Nfix expressing BaF/3 cells over 5 days in culture, showing mean of 3 technical replicates +/- SD. Graph is representative of 2 independent experiments. (D) Graph of 5 days of cell growth of GFP sorted BaF/3 cells transduced with either MigR1 or Nfix. Each point represents the average number of cells from 2 independent experiments. Error bars denote +/- SD. (E) Tracking Baf/3 cell division after 5 days in culture as shown by dilution of Cell Trace Violet in GFP expressing cells. MigR1 and Nfix overexpressing cells are compared to unstained control and Day 0 cells as positive and negative controls respectively. Graph is representative of 2 independent experiments. (F) Representative FACs plot of the expression of apoptotic markers AnnexinV and DAPI in MigR1 or Nfix transduced, GFP sorted BaF/3 cells, 4 days after transduction. (G) Graph of the average percentage of GFP sorted BaF/3 cells expressing DAPI, AnnexinV and live cells (double negative for DAPI and AnnexinV), from 2 independent experiments. Error bars denote +/- SD. (TIF) [file pone.0120102.s002.tif]
