# Supplementary material for: Nfix Expression Critically Modulates Early B Lymphopoiesis and Myelopoiesis
Source: PLoS One. 2015 Mar 17;10(3):e0120102. doi: 10.1371/journal.pone.0120102 (PMC4363787; doi:10.1371/journal.pone.0120102)
Supplement: S1 Table — (PDF) [file pone.0120102.s003.pdf]

S1 Table

Sorting stem and progenitor populations

Population	Identification	Usage	Figure
hCD25- CLP<br>$hCD25^+$ CLP<br>Pro B cells<br>Pre B cells<br>Mature B cells	$Lin^-B220^-CD19^-IL7R^+Flt3^+Sca1^{low}Kit^{low}CD25^-$<br>$Lin^-B220^-CD19^-IL7R^+Flt3^+Sca1^{low}Kit^{low}CD25^+$<br>$CD19^+AA4.1^+CD43^{low}$<br>$CD19^+B220^+CD43^-IgM^-$<br>$CD19^+IgM^+$	Analysis of GSE11110 (Mansson et al., 2008)	Fig 3A
Pre-pro B<br>Pro-B<br>Pre-B<br>Immature B	$B220^+CD43^+CD19^-IgM^-$<br>$B220^+CD43^+CD19^+AA4^+$ IgM^-<br>$CD43^-B220^+IgM^-CD19^+$<br>$CD43^-B220^+CD19^+IgM^+$	Cell Sorting	Fig 3B
HSC2<br>Early B-cell<br>Pro-B cells	$CD38^-CD34^+$<br>$CD34^+CD10^+CD19^+$<br>$CD34^-CD10^+CD19^+$	Analysis of GSE24759 (Novershtern et al., 2011)	Fig 3C
HSC<br>LMPP<br>CMP<br>GMP<br>MEP	Lin-cKit+Sca1+Flt3-<br>Lin-cKit+Scat1+Flt3+<br>Lin-cKit+CD34intCD16/32int<br>Lin-cKit+CD34+CD16/32+<br>Lin-cKit+CD34-CD16/32-	Cell Sorting	Fig 3D<br>Fig 5C-D
HSC	Lin-cKit+Sca1+Flt3-	Cell Sorting	Fig 4B
