# Supplementary material for: Nfix Expression Critically Modulates Early B Lymphopoiesis and Myelopoiesis
Source: PLoS One. 2015 Mar 17;10(3):e0120102. doi: 10.1371/journal.pone.0120102 (PMC4363787; doi:10.1371/journal.pone.0120102)
Supplement: S2 Table — (PDF) [file pone.0120102.s004.pdf]

S2 Table

FACs Antibodies (eBioScience)

Marker	Clone
anti-Gr-1	(RB6-8C5),
anti-CD11b	M1/70
anti-B220	RA3-6B2
anti-CD19	ID3
anti-sIgM	11/41
anti-CD43	R2/60
anti-AA4.1	AA4.1
anti-CD4	RM4-5
anti-CD8a	53-6.7
anti-CD45	30-F11
anti-cKit	2B8
anti-Sca1	D7
anti-Flt3	A2F10
anti-CD34	RAM34
anti CD16/32	93
anti-IL7Ra	A7R34
