# Supplementary material for: Nfix Expression Critically Modulates Early B Lymphopoiesis and Myelopoiesis
Source: PLoS One. 2015 Mar 17;10(3):e0120102. doi: 10.1371/journal.pone.0120102 (PMC4363787; doi:10.1371/journal.pone.0120102)
Supplement: S3 Table — (PDF) [file pone.0120102.s005.pdf]

S3 Table

Primer Sequences (rtPCR & Fluidigm)

Gene Symbol		Oligonucleotide sequence (5'-3')
**Abl**	*forward*	TGGAGATAACACTCTAAGCATAACTAAAGGT
	reverse	GATGTAGTTGCTTGGGACCCA
**Gusb**	*forward*	GGGACAAAAATCACCCTGCG
	reverse	GCGTTGCTCACAAAGGTCAC
**B2M**	*forward*	TGCTATCCAGAAAACCCCTCA
	reverse	TTTCAATGTGAGGCGGGTGG
**Tbp**	*forward*	GCAGTGCCCAGCATCACTAT
	reverse	GCCCTGAGCATAAGGTGGAA
**Hprt1**	*forward*	GAGAGCGTTGGGCTTACCTC
	reverse	ATCGCTAATCACGACGCTGG
**Rnf20**	*forward*	ACCATCAATGCCCGGAAGTT
	reverse	GCAGCGATACTCTGGGGTTT
**Enox2**	*forward*	CGGCTGTTTAAGGAGTAGCG
	reverse	AGCTGGTTCAATCCTGGAACAT
**Nfix**	*forward*	AAACCAGCAAGGAGATGCGG
	reverse	TGATGCAGTCGCAACTGGAG
**Blink**	*forward*	GGTAAGCCCTGGTATGCTGG
	reverse	CTAGGGTGTACGGCTGCTTG
**E2a**	*forward*	GCCTGGATACTCAGCCGAAG
	reverse	TAGAAGGGGGAGGGGTAAGC
**Pax5**	*forward*	GAC ATC TTC ACC ACC ACG GAA
	reverse	AGGACTGTGGGCCTGGAAC
**Ikaros**	*forward*	AGGGTCAAGACATGTCCCAAG
	reverse	GCTGTGCTCCAGAGGTAGTG
**CD19**	*forward*	AAACCTGACCATCGAGAGGC
	reverse	GGGTCAGTCATTCGCTTCCTT
**Flt3**	*forward*	TTGGCCTTTGTGTCTTCCGT
	reverse	TTGCGAGCTGGTAGCGTTTA
**Ebf1**	*forward*	CCATGTCCTGGCAGTCTCTGA
	reverse	TCCATCCTTCACTCGGGCT
**Id1**	*forward*	GAACCGCAAAGTGAGCAAGG
	reverse	AACACATGCCGCCTCGG
**Id2**	*forward*	CCTGGACTCGCATCCCACTA
	reverse	AGGGAATTCAGATGCCTGCAA
**Id3**	*forward*	CCTCTTAGCCTCTTGGACGAC
	reverse	CAGCTGTCTGGATCGGGAGAT
**Pu1**	forward	CGCACGAGTATTACCCCTAT
	reverse	GAGCTCCGTGAAGTTGTTCT
**Mpo**	*forward*	CCCAGGCATAAAAACCCGT

	reverse	TGAATTCTCCACTTCCCCCAG
**Mmp9**	*forward*	ACGACATAGACGGCATCCAG
	reverse	TGGGACACATAGTGGGAGGT
**C/ebpe**	*forward*	GAGGCAGCTACAATCCCCTG
	reverse	CACAGGGGCCTTGAGGACA
**Notch1**	*forward*	ATCAAGCGCTCTACAGTGGG
	reverse	AAACCTGACCATCGAGAGGC
**Gata3**	*forward*	GGGTCAGTCATTCGCTTCCTT
	reverse	GTTCACACACTCCCTGCCTT
**CD3delta**	*forward*	GGAACACAGCGGGATTCTGG
	reverse	ACCATCCTTCCACCGTTCCA
**CD3gamma**	*forward*	TCTCATTGCGGGACAGGATG
	reverse	TATTCCCGGTCCTTGAGGGG
**CD7**	*forward*	CACCTGGATTTGGGCGTCAT
	reverse	ACTGGTGTACGTCTTGGGC
**Rictor**	*forward*	GAGGTGGAGAGGACACAAGC
	reverse	CCGGACCATTCTGTCTCGTT
**Gata1**	*forward*	CTCCCCAGTCTTTCAGGTGT
	reverse	CAGGGTAGAGTGCCGTCTTG
**Gata2**	*forward*	TCACCCCTAAGCAGAGAAGC
	reverse	CATTGCACAGGTAGTGGCCC
**Fog1**	*forward*	CAAACTTCCTCCAGACAGCC
	reverse	CTCTGGTCTCTCCGTTGGTG
**Klf1**	*forward*	CTAAGAGGCAGGCGGCACAT
	reverse	CTGAGCGAGCGAACCTCC
**Fli1**	*forward*	ATCTGAAGGGGCTACGAGGT
	reverse	ACCACAGACAGAGCCTCCTTA
**Mpl**	*forward*	AACAAGACCGCACTAGCTCC
	reverse	GCGGTTCCTCCTCTTCACAT
**Trfr1**	*forward*	TCCGCTCGTGGAGACTACTT
	reverse	ACATAGGGCGACAGGAAGTG
